# Unconscious bias in students of health professions – an experimental vignette study

**DOI:** 10.1186/s12909-025-08317-x

**Published:** 2025-12-10

**Authors:** Ursula Meidert, Marc Höglinger, Frank Wieber, Andreas Gerber-Grote

**Affiliations:** 1 School of Health Sciences, Zurich University of Applied Sciences, Katharina-Sulzer- Platz 9, Winterthur, 8400 Switzerland; 2 School of Management and Law, Zurich University of Applied Sciences, Winterthur Institute of Health Economics, Gertrudstrasse 8, Winterthur, 8400 Switzerland; 3 Department of Psychology, University of Konstanz, Konstanz, Germany

**Keywords:** Unconscious bias, Health disparity, Health care, Students, Education

## Abstract

**Background:**

Unconscious bias refers to automatic, implicit attitudes or stereotypes that influence our understanding, decisions, and actions without our conscious awareness. It is recognised as a significant problem in healthcare contributing to disparities in treatment. To date it remains unclear how unconscious bias towards patients develops among health professionals. One hypothesis is that such bias is acquired during education, either through teaching content or by observing other health professionals interacting with patients and adopting their behaviour. We investigated whether health care students show an unconscious bias and whether there are indications that it develops during their professional education.

**Methods:**

We conducted a factorial survey experiment with bachelor’s and master’s students enrolled in various health professions’ programmes. Unconscious bias was assessed using three written vignettes describing clinical situations involving patients. Participants were asked to evaluate their likelihood of helping immediately, expected patient adherence, and expected quality of the patient relationship. Vignette dimensions contained common sources of bias that were experimentally manipulated: gender, age, socio-economic status, migration status, diagnosis (physical or mental illness), and sexual orientation. Multivariable regression models were used to estimate the causal effects of patient characteristics on vignettes on students’ responses. Additionally, an Implicit Association Test (IAT) on unconscious bias regarding homosexuality was used to measure implicit bias. Explicit attitudes were assessed via self-report.

**Results:**

A total of 470 students (response rate 21.5%) participated. Vignette analysis showed no differences in stated helping intention, adherence expectations, or relationship assessments with regard to patient characteristics such as gender, age, socio-economic status, foreign name, sexual orientation or diagnosis. No systematic differences were observed for subgroups of participants such as year of study, programme type, prior work experience, or reported exposure to bias behaviour by health care staff. Similarly, IAT results indicated no overall bias towards homosexuality.

**Conclusions:**

We found no evidence of systematic unconscious bias among students’ helping intentions, expected patient adherence, and expected patient relations across various patient characteristics. Comparisons across study years and programs provided no indication that such biases emerge or intensify during training. If replicated, these results would be encouraging, as it indicates an absence of unconscious bias in health care students.

**Supplementary Information:**

The online version contains supplementary material available at 10.1186/s12909-025-08317-x.

## Background

Over the last two decades, research has shown that health professionals do not treat their patients impartially but exhibit unconscious bias towards them [1–5]. A recently conducted scoping review identified numerous studies in which health professionals demonstrated unconscious bias towards patients. These biases included gender, age, ethnicity/race, socio-economic status, weight or body morphology, and health conditions such as HIV/AIDS, mental health disorders, or disability [3].

Research has shown that unconscious bias not only contributes to health disparities but can also influence clinical decision-making and patient care. For example, studies have found that unconscious bias affects the prescription of pain medication [6], waiting times for assessments or treatments [7], attribution of back pain [8], the number of referrals to specialists or diagnostic assessments [9], and can lead to unintentional discrimination [10]. Unconscious bias also impacts verbal and non-verbal communication with patients [11], such as the number of questions asked, verbal dominance in consultations, and reduced patient-centred or supportive communication [12]. This, in turn, can affect patients’ perception of care [11].

The literature differentiates between explicit and implicit (or unconscious) bias. An explicit bias refers to conscious thoughts and beliefs that an individual can report [13]. An implicit or unconscious bias, on the other hand, is not accessible to conscious awareness. It is often described as the result of involuntary associations or attitudes that influence perception, behaviour, decision-making, and social interactions outside of conscious control [2, 5, 14–16]. These unconscious associations or attitudes are often manifested in our non-verbal behaviours, such as eye contact, physical proximity [2] or chosen seating distance [17].

A person’s explicit and implicit biases may differ or even contradict one another. For example, individuals may consciously reject negative stereotypes about disadvantaged groups in self-reports [5], while still exhibiting bias in reaction time-based measures [2, 18] such as the Implicit Association Test (IAT) [19] or the Go/No-Go Association Task (GNAT) [20]. Green et al. [21], for instance, found that physicians who did not explicitly report negative bias against African American patients and did not classify them as less cooperative nonetheless exhibited implicit beliefs that these patients were less cooperative than white patients. Implicit and explicit biases can be treated as distinct constructs [22].

Despite the increasing attention to unconscious bias in health care, it’s still unclear how unconscious biases are obtained. Some scholars suggest that unconscious bias among health professionals mirrors that of the general population [2] and thus represent shared dominant cultural stereotypes or prejudices [23]. Others suggest that such biases might seep in through professional formation and training. For example, teaching methods like clinical vignettes may unintentionally reinforce bias [15, 16]. Furthermore, observing supervisors or more senior health care staff interact with minority patients or overhearing derogatory remarks contribute to increased bias among students of health professions [24], consistent with social learning theory [25].

Other researchers propose that unconscious bias stems from assumptions derived from prevalence rates [15, 23] or from “statistical discrimination”, in which health care professionals might have a harder time interpreting test results for minorities [26, 27]. In the absence of a clear diagnosis, clinicians may rely more on perceived group-based probabilities, potentially leading to a poor match between the patients’ needs and the care provided [23]. Together, although several pathways for the formation of unconscious bias in health professionals have been proposed, it is still not well understood.

Additionally, previous research on unconscious bias has been somewhat selective. The majority of studies have focused on physicians and nurses, predominantly examined racial bias, and were largely conducted in North America. In contrast, there is limited research addressing other health professional groups, such as occupational therapists, physiotherapists or midwifes [3]. Similarly, evidence on forms of bias beyond race remains limited and at times, inconsistent. Research conducted in European contexts is also scarce, despite that cultural, societal, and historical differences may produce divergent findings. For example, Khosla et al. [28] found that French physicians did not show bias towards Black patients, unlike their US counterparts. Moreover, while most existing studies focus on a single dimension of bias, little attention is given to intersecting identities such as gender and age or migration background and sexual orientation [4].

Therefore, this study has several aims: to contribute to the understanding of unconscious bias in a European health care context; to examine the role of professional training in the development of such bias; and to investigate intersecting identities in relation to unconscious bias. The following research questions guide this study:

- Do students in the healthcare professions exhibit implicit or explicit bias towards patients?
- Which biases are most prevalent, and in which professional group?
- Do intersecting patient characteristics amplify unconscious bias among health care students?
- Do unconscious biases differ across stages of professional training?
- Do students who received training on unconscious bias exhibit less pronounced unconscious bias than those who did not?

## Method

A factorial survey [29, 30] was conducted online with students of health professions to assess the presence of unconscious biases (e.g., [31, 32]). Participants were presented with so-called “vignettes” describing situations in which patients required help, and were asked to indicate their helping intentions. Vignette dimensions such as patient gender, age, diagnosis type, presumed origin (domestic or foreign), socio-economic status, and sexual orientation were randomly varied to assess their effect on students’ helping intentions.

In addition, an Implicit Association Test (IAT) measuring unconscious bias towards homosexuality was administered. The IAT is widely used in research on implicit biases, especially in areas like racism, sexism, and prejudice [19] (for further information see: https://implicit.harvard.edu/implicit). It is a psychological assessment designed to measure the strength of automatic associations between concepts (e.g. homosexuality/heterosexuality) and evaluations (e.g. good/bad) based on participants’ reaction times during categorisation tasks.

Ethical approval was obtained by the Ethics Review Board of the Zurich University of Applied Sciences (No. EA-ZHAW 2024-008-G). 

### Participants and procedures

Participants were recruited from the School of Health Sciences at Zurich University of Applied Sciences. All bachelor’s and master’s students in the nursing, physiotherapy, occupational therapy, midwifery and health promotion and prevention programmes (*N* = 2,176) were invited to participate.

Data collection occurred in two phases: in June 2024 at the end of the spring semester for students in semesters 2–6 and in September 2024 for students beginning their first semester. Students received an email invitation to their personal mail accounts, followed by a reminder after two weeks. Additionally, the study was promoted via the university’s internal student portal, social media, flyers distributed on campus, and brief in-class announcements (where possible). As incentive, there was a raffle of three CHF 150 gift vouchers among participants.

### Study measures

#### Vignettes

For the present study, scenarios, so-called “vignettes” were designed to resemble brief, realistic situations, accessible even to first-semester students. Scenarios had to be applicable across different health professions; therefore, they were kept general, avoiding tasks that would normally fall only within the remit of one specific profession. 

The vignettes were developed in consultation with faculty members from the relevant disciplines (nursing, midwifery, physiotherapy, occupational therapy, and health promotion and prevention). Three scenarios were created, depicting students working part-time in a clinical setting. This is common among our student population. Each vignette was presented only once, kept brief to minimize cognitive load, an began with: “You work part-time as an assistant in a hospital…”.

To test for unconscious biases, we varied gender, socio-economic status, age, migration background, diagnosis type (physical or mental illness), and sexual orientation of the patients depicted in the vignettes. Each patient was briefly described, e.g.:

Mr./Mrs. Hafner/Kumarsami is 29/63 years old, lives in the region, works as a financial specialist/temporary help in the insurance sector and is hospitalized dues to a physical/mental illness.

A full factorial design was used for two scenarios, resulting in 64 possible combinations of patient characteristics. In the third scenario, the diagnosis variable was removed to avoid implausible combinations, yielding 32 possible combinations. All questions (see Table 1) where answered on 6-point Likert scales (1 = very unlikely to 6 = very likely), except for the pleasantness item, which used a 5-point Likert scale (1 = very unpleasant to 5 = very pleasant with 3 = neutral midpoint).

**Table 1:** Vignette questions

Vignette 1
1. Intention to help during break – How likely are you to postpone your break and help immediately?
2. Expected patient acceptance of help – How likely is the patient to accept your support?
3. Expected relationship quality – How likely is it that you will build a good relationship with the patient?
4. Expected pleasantness – How pleasant do you find the task of supporting the patient?
Vignette 2
1. Expected patient adherence in sitting watch – How likely is it that the patient will follow your instructions to remain in bed?
2. Expected relationship quality in sitting watch – How likely is it to build a good relationship with the patient?
3. Perceived pleasantness of sitting watch – How pleasant do you find supporting the patient in this task?
Vignette 3
1. Intention to call for support – How likely are you to call your colleague back from the break?

To evaluate the perceived realism of the vignettes, participants were also asked to rate how realistic they perceived the presented scenarios using a 5-point Likert-scale (1 = unrealistic to 6 = realistic).

#### Implicit association test

In addition to the vignettes an Implicit Association Test (IAT) [33] was included at the end of the questionnaire to assess implicit attitudes toward homosexuality. The IAT used in this study was based on previously used materials for IAT for homosexuality that was freely available from Minno Suite (https://minnojs.github.io/docs/) on GitHub (https://github.com/baranan/minno-tasks/tree/master/IAT). Stimuli included both words and images, and the task compared reaction time for positive and negative associations. The results are translated into the d-score ranging from − 2 to + 2: scores >0.15 indicate a slight preference, >0.35 a moderate preference, and >0.65 a strong preference. In this test positive scores implied a preference for homosexuality, while negative scores represent a preference for heterosexuality or a bias towards homosexuality. The IAT lasts about 5–7 min and was administered only once, testing a single bias dimension at the end of the survey.

#### Explicit bias and familiarity

Measuring both unconscious and conscious attitudes is considered a comprehensive approach to explore attitudes and stereotypes that influence behaviour towards patients solely [34]. Therefore, participants rated their warmth or coldness towards people representing the same bias dimensions used in the vignettes (gender, socio-economic status, age, migration background, diagnosis type (physical or mental illness), and sexual orientation). The original 11-point scale (0 = unsympathetic, 5 = neutral, 10 = sympathetic) was transformed so that the neutral midpoint corresponded to 0, resulting in a range from − 5 (unsympathetic) to + 5 (sympathetic). Participants rated attitudes towards specific target groups representing each dimension, such as male and female individuals, people with high or low socio-economic status, young or elderly adults, migrants and Swiss nationals, individuals with physical or mental illnesses, and heterosexual or LGBTQ + persons. These ratings enabled comparison between implicit and explicit attitudes. Additionally, participants were asked about their familiarity with individuals from these groups, based on the assumption that greater familiarity might reduce explicit bias. To compare attitudes towards different groups within the same participants, Wilcoxon signed-rank tests for paired samples were conducted.

#### Social desirability and social learning

Given the topic’s sensitivity, the short version of the Social Desirability-Gamma Scale (KSE-G) [35] was included to measure possible response bias. Scores were calculated following the method described by Kemper et al. resulting in two subscales: *exaggeration of positive qualities* (PQplus) and *understatement of negative qualities* (NQminus). To explore the potential influence of social learning, students were asked whether they had witnessed discrimination against patients by staff or supervisors. If they clicked “yes”, an open-ended field appeared prompting them to briefly describe the situation.

#### Demographic variables

Demographic questions included gender identity, age, nationality, sexual orientation, affluence of household, and parental education level. Other variables were study programme, current semester, professional work experience, experience with diversity or unconscious bias training, personal attitudes towards equal treatment of patients, and identification with their (future) profession.

#### Survey tool

The survey was administered using Unipark© by Tivian©. Vignette texts were presented as running text, and the programme randomized vignette assignment. The randomization function was tested multiple times to ensure proper functionality.

#### Pre-test

The questionnaire was pre-tested in two stages. First, five faculty members reviewed the items to ensure that the vignettes were applicable across all professions and the answer categories were complete. Second, two nursing classes (*N* = 30) at the Center for Training in Healthcare ZAG in Winterthur, Switzerland who were not part of the target sample participated in a trial run. The pre-test confirmed the suitability of the vignettes, and no technical problems occurred. The data showed approximately normal distribution.

#### Statistical analysis

Statistical analysis was performed using Stata© 18. The effects of the patient characteristics on responses to vignettes were estimated using multivariable linear regression. Subgroup differences in effects were assessed using an F-test. For the IAT we used the scorer programme on GitHub. The d-score calculation follows Richetin et al. [36] recommendations for calculating the d-score.

## Results

### Participation

A total of 470 students participated in the study (participation rate = 21.5%). After data cleaning and removing 36 so-called “speeders” (faster than 3 min) and 12 participants who did not finish the questionnaire 422 participants remained in the sample. Participating students roughly correspond to the total study population in terms of gender and programme type.

### Participants’ characteristics and key variables

Participants’ characteristics are summarised in Table 2. The largest group of participants was from Physiotherapy (109; 25.8%), followed by Nursing (99; 23.5%), Occupational Therapy (80; 19.0%), Health Promotion & Prevention (70; 16.6%), and Midwifery (64; 15.2%). Regarding the study year, 201 participants (47.6%) were in the first semester of a Bachelor’s programme, while 221 (52.4%) were in later semesters of a Bachelor’s programme or enrolled in a Master’s programme. The regular duration of studies is six semesters for full-time and nine semesters for part-time Bachelor’s students, and four semesters for full-time and six semesters for part-time Master’s students. Most participants (88.2%; *n* = 372) were enrolled in a Bachelor’s programme, while a minority attended a master’s programme (11.8%; *n* = 50). The mean age of respondents was 25.2 years, with a range from 17 to 57 years. Of the participants, 91.5% (*n* = 386) identified as female, 7.6% (*n* = 32) as male, and 0.9% (*n* = 4) as non-binary or other. Regarding nationality, 318 participants (75.4%) were Swiss, 68 (16.1%) had Swiss and a second nationality, and 36 (8.5%) had another nationality. In terms of self-reported family income, 14.0% (*n* = 59) reported very low to low income, 57.3% (*n* = 242) middle income, 23.9% (*n* = 101) high to very high income, and 4.7% (*n* = 20) did not report or did not know. Most participants identified as heterosexual (84.1%; *n* = 355), 12.1% (*n* = 51) as non-heterosexual (homo- or bisexual), and 3.8% (*n* = 16) did not report or did not know.

**Table 2:** Characteristics of participants (*N* = 422)

Characteristics	*n*	%
*Health Profession*		
Physiotherapy	109	25.8
Nursing	99	23.5
Occupational Therapy	80	19.0
Health promotion & prevention	70	16.6
Midwifery	64	15.2
*Semester*		
1 st semester BSc	201	47.6
2nd + Semester BSc and more advanced students	221	52.4
*BSc/MSc*		
Bachelor student	372	88.2
Master student	50	11.8
*Age*		
< 20–23	221	52.4
24+	201	47.6
*Gender identification*		
Female	386	91.5
Male	32	7.6
Other and not reported	4	0.9
*Nationality*		
Swiss	318	75.4
Swiss and 2nd nationality	68	16.1
Other nationality	36	8.5
*Family income (self-reported)*		
Very low – low	59	14.0
Middle	242	57.3
High – very high	101	23.9
Not reported/don’t know	20	4.7
*Sexual orientation*		
Heterosexual	355	84.1
Non-heterosexual (Homo- & Bisexual)	51	12.1
Not reported/don’t know	16	3.8

Table 3 shows students self-reported experiences. Of the 422 students, 41.7% (*n* = 176) reported having had no input on unconscious bias in class before, 48.8% of students (*n* = 206) did either have a brief input (30.1%; *n* = 127) or more in-depth input (18.7%; *n* = 79) while 6.2% (*n* = 26) were not sure, and for 3.3% (*n* = 14) the question did not apply. We also asked whether students had an unconscious bias training, which applied to 12.3% (*n* = 52) of students and 82.2% (*n* = 347) it did not apply, while 4.7% (*n* = 20) were not sure, and for 3 (0.7%) students the answer was missing. When asked if they manage to treat all patients equally in practice, a majority of 74.9% (*n* = 316) of students indicated that they do so *often* or *always*. A further 16.1% (*n* = 68) stated *sometimes*, while 6.2% (*n* = 26) reported seldom to never. Some 2.8% (*n* = 12) of students didn’t have any patient contact yet. Most students reported identifying either *very strongly* (17.3%; *n* = 73) or *strongly* (54.0%; *n* = 228) with their chosen profession. A further 26.3% (*n* = 111) identified *somewhat*, while 2.4% (*n* = 10) reported that they *rather did not identify* with their profession.

**Table 3:** Participants’ Self-Report on key variables (*N* = 422)

Characteristics	*n*	%
*Unconscious bias addressed in class*		
Yes	206	48.8
No or don’t know	216	51.2
*Bias Training*		
Bias training	52	12.3
No bias training	370	87.7
Professional experience		
None	12	2.8
Up to 1 year	175	41.5
More than 1 year	235	55.7
*Witnessed discrimination*		
Yes	210	49.8
No/don’t know	212	50.2
*Equal treatment of patients*		
Always or often	316	74.9
Sometimes	68	16.1
Seldom or never	26	6.2
No patient contact yet	12	2.8
*Identification with profession*		
Very strongly	73	17.3
Strongly	228	54.0
Somewhat	111	26.3
Rather not	10	2.4

### Perceived realism of vignettes

A majority of students perceived the vignettes as realistic: 28.4% (*n* = 118) rated them as *realistic*, and 46.2% (*n* = 192) as *rather realistic*. A further 21.6% (*n* = 90) considered them *partially realistic*, while 2.9% (*n* = 12) rated them as *rather unrealistic*, and 0.9% (*n* = 4) as *unrealistic*.

### Implicit bias: results of vignettes

**Fig. 1:**
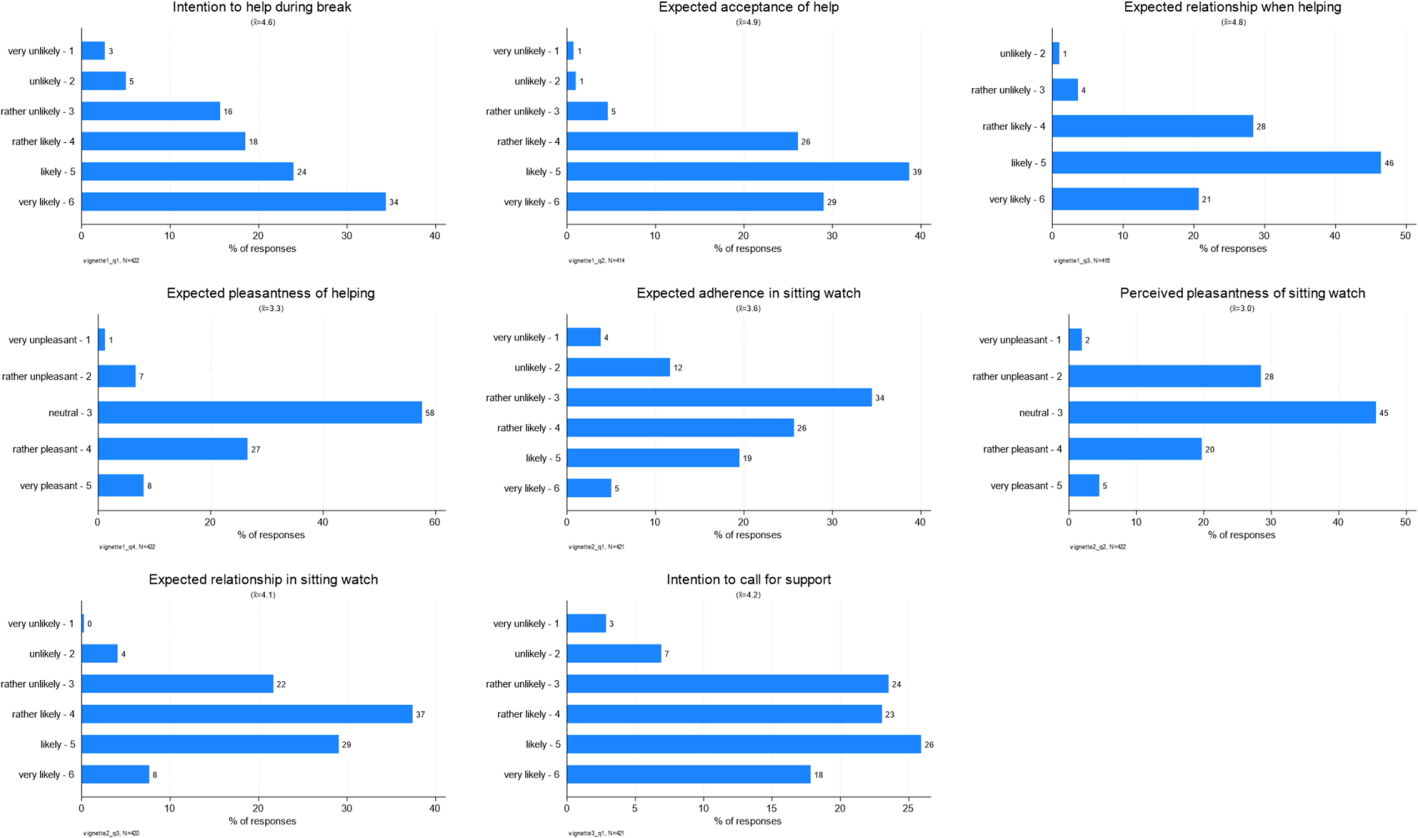
Distribution of vignette assessment

Students’ assessments of the vignettes revealed a high inclination to help patients, a high expectancy of patient cooperation, and a high task pleasantness. Figure 1 displays the overall distribution of responses. Across all vignettes and outcomes, mean scores were skewed toward the positive end of the scales, suggesting favourable expectations regarding patient behaviour and the quality of interaction.

**Fig. 2:**
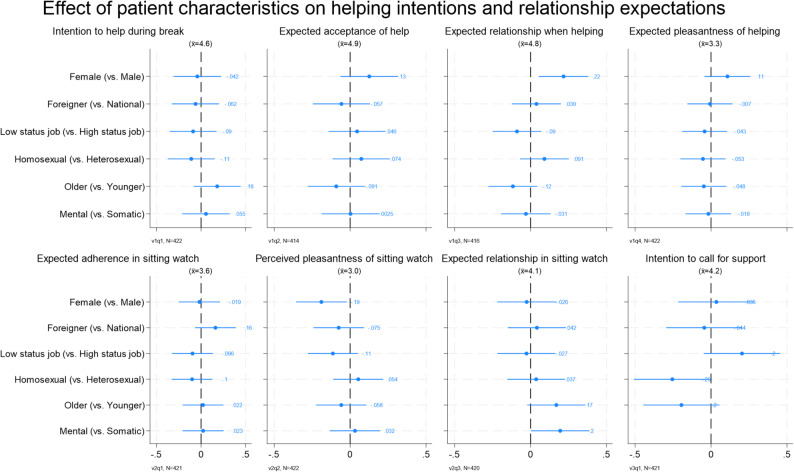
Effect of patient characteristics on vignettes’ assessments. Estimates with 95%-CI. $\overline{\mathrm{x}}$ stands for the sample mean

To investigate the potential influence of patient characteristics on these assessments, we conducted regression analyses for each vignette outcome (see Fig. 2). Overall, the analyses revealed no consistent or substantial evidence of bias regarding patient characteristics such as gender, age, socioeconomic status, migration background, diagnosis type (mental vs. somatic), or sexual orientation. Only a few isolated and small effects reached statistical significance. These included a more positive assessment for female (vs. male) patients regarding the *expected relationship when helping*, a slightly more negative evaluation of the *expected relationship in sitting watch* for female (vs. male) patients, a marginally lower *intention to call for support* for homosexual (vs. heterosexual) patients, and a slightly more favourable rating of *task pleasantness in sitting watch* for patients with a mental (vs. somatic) condition. However, these effects did not follow a consistent pattern across the different vignettes or patient characteristics. In addition, due to the multiple testing, for which we do not correct, these barely statistically significant effects are likely due to random error.

**Fig. 3:**
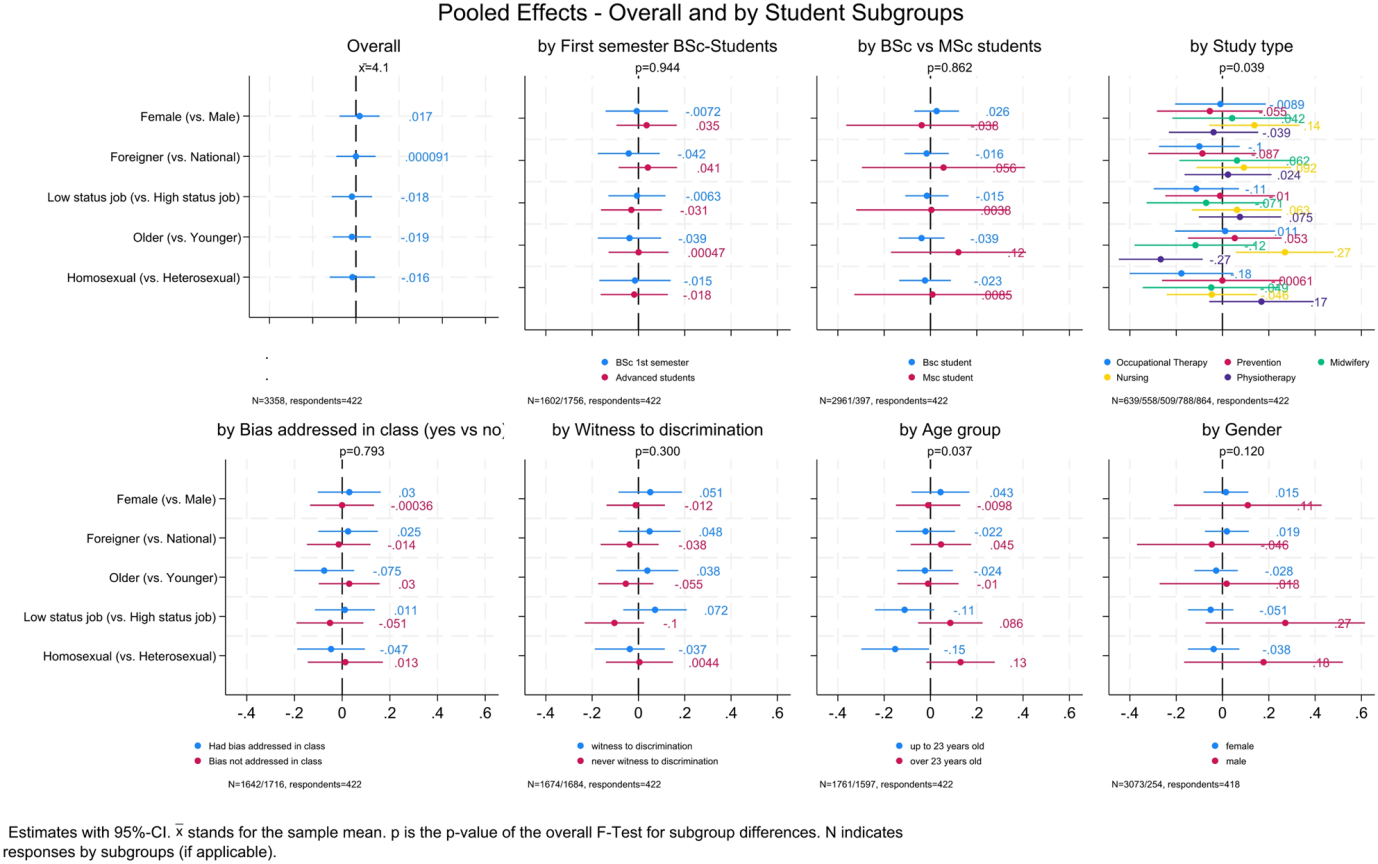
Pooled vignettes’ assessment overall and by student subgroups

To assess the robustness of these findings, we pooled all vignette outcomes and analysed them jointly (Fig. 3, top left panel). Again, no meaningful effects or statistically significant results were observed for any patient characteristic. Stratified analyses by student subgroups (e.g. age, gender, semester, professional background, exposure to training or observed bias) also revealed no consistent patterns of differential assessment. An exception was a small significant effect for students aged 23 or younger, who rated *homosexual patients* slightly less favourably on the *intention to call for support* outcome while students older than 23 years showed a comparably sized but positive effect for the same group and outcome. Among professional subgroups, we observed two isolated significant effects: physiotherapy students rated elderly patients slightly more negatively, while nursing students showed a small positive bias towards this group.

In a final step, we tested for intersectionality effects by analysing combinations of patient characteristics (see Fig. 4 in the supplementary material). We hypothesised that particular combinations, such as female patients of low socioeconomic status with a migration background, might elicit more negative assessments than each characteristic individually. However, the analysis showed no significant deviations from the average for any combination, with one exception: female, Swiss-national patients with high-status occupations received slightly more positive ratings on *expected relationship when helping*.

In summary, across individual, pooled, and interactional analyses, there was no compelling evidence for systematic bias in students’ assessments of patients (See Fig. 5 in the supplementary materials). While some isolated effects emerged, they were not consistent in direction or strength, and did not support the hypothesis that unconscious bias was present in students’ decision-making in this vignette-based experiment.

### Implicit bias: implicit association test

A total of 370 students completed the IAT for homosexuality. For 14 participants, IAT data could not be linked to the survey response and had to be excluded from further analyses. Among the remaining students, 84 students (23.6%) showed a preference for homosexuality, 102 students (28.7%) sowed no preference, and 170 (47.8%) students had a preference for heterosexuality. The mean was − 0.11 (min − 1.05, max.+0.97) indicating no automatic preference for heterosexuality or homosexuality and therefore no implicit bias. There were no differences between first semester BSc students (*n* = 179) and students from higher semesters or master students (*n* = 177), nor between students (*n* = 193) with work experience compared to students with only little or no work experience (*n* = 163), nor was there a difference between gender of students. There was also no difference between students who attended any bias training or covered the subject in class and those who did not. However, there was a statistically significant difference in sexual orientation (Chi-Squared-Test, *p* < 0.001). Students who indicated being heterosexual (*n* = 299) had a mean d-score of − 0.16 which is considered as a slight preference towards heterosexuality. Whereas students indicating being homosexual, bisexual or “other” (*n* = 45) had a mean d-score of + 0.16 which is considered a slight positive preference towards homosexuality.

### Explicit bias and familiarity

Almost all students rated selected population groups as either *neutral* or *sympathetic* on a continuous 11-point scale ranging from *unsympathetic (0)*, through *neutral *(See Fig. 5 in the supplementary materials) , to *sympathetic* (See Fig. 10 in the supplementary materials). Mean ratings for each target group (e.g. people with mental illness, people of foreign nationality) are reported in Table 4., Wilcoxon signed-rank tests for paired samples revealed statistically significant differences (*p* < 0.001) between all group pairs except for people with a foreign nationality compared to those with Swiss nationality.

Mean sympathy ratings correlated with the amount of personal contact students reported having with the respective group in their daily lives, as measured by Spearman’s rho (non-parametric correlation). This pattern held for all group comparisons except for those involving people with physical or mental illnesses, where the correlation was not statistically significant.

**Table 4:** Explicit bias

Group	Mean	SD	Min	Max	Correlation Coefficient	*P* value
Elderly women (65+)	2.41	1.96	−4	5	0.18	< 0.001
Elderly men (65+)	1.65	2.05	−5	5	0.18	< 0.001
People with mental illness	1.12	1.84	−3	5	0.26	< 0.001
People with physical illness	1.84	1.89	−2	5	0.07	n.s.
People of foreign nationality	1.86	2.05	−3	5	0.22	< 0.01
People of Swiss nationality	1.87	1.96	−3	5	0.42	< 0.01
Heterosexual men	1.62	2.15	−5	5	0.17	< 0.01
Homosexual men (gay)	2.01	2.03	−4	5	0.13	< 0.01
Heterosexual women	2.15	2.0	−4	5	0.11	< 0.05
Homosexual women (lesbian)	1.93	2.07	−5	5	0.23	< 0.01
People considered financially rich	0.7	2.2	−5	5	0.25	< 0.01
People considered financially poor	1.58	1.92	−3	5	0.16	< 0.01

### Social desirability

Social desirability scores for : *exaggeration of positive qualities* (PQplus) was 2.8, and for *understatement of negative qualities* 0.7. Both values are consistent with the reference scores reported by Kemper et al. [35].

## Discussion

### Main findings and interpretation

The aim of this study was to examine whether health profession students in Switzerland show at group level implicit or explicit biases towards patients, which biases are most prevalent and whether intersecting patient characteristics amplify unconscious bias. We also explored the potential contribution of professional training to such biases and which theoretical perspective on their development is more plausible.

To address these questions, we combined two established approaches: a factorial vignette design manipulating key patient characteristics (e.g. gender, age, socio-economic status, migration background, diagnoses, and sexual orientation) and an Implicit Association Test for sexual orientation.

Results from the vignette method showed no systematic differences in helping intention, expected adherence or expected patient relationship regarding key patient characteristics. Hence, there appears to be no group-level systematic bias with regard to these analysed characteristics. These results do not differ by subgroup of respondents, such as type of study programme, level of training, or prior professional experience. Bias training or prior exposure to biased behaviour by others also showed no impact/effect. Only small differences emerged in isolated instances. Given the number of subgroup comparisons conducted and the absence of systematic trends, these findings are likely due to chance and do not support the presence of subgroup-specific bias. Our exploration of intersecting identities revealed isolated effects (e.g. the combination of female gender and foreign background), but no consistent pattern across scenarios. Note that the power for this analysis is rather weak. Results from the IAT showed no preference between heterosexual or homosexual individuals, indicating that, within this cross-sectional sample, no implicit bias could be found.

Although we hypothesized that unconscious biases might be acquired during health professions education, the lack of detected bias in this cross-sectional sample does not allow conclusions about the timing or development of such biases. Future longitudinal studies are needed to address this question.

Explicit bias measure suggest that students tend to express generally positive feelings towards all groups. Feelings of sympathy correlated with the amount of contact students have with the respective group which – according to the intergroup contact theory [37] – is expected only under supportive conditions [38]. Interestingly, they reported more sympathetic attitudes towards minority groups, such as individuals with a migrant background or those from lower socio-economic backgrounds, compared to Swiss nationals or individuals with a high socio-economic status. These results are noteworthy in comparison with similar studies, which often report more neutral or even negative attitudes towards marginalised groups (e.g., [39]).

### Comparison to existing literature

Our findings diverge from several previous studies reporting unconscious bias among students of health professions. For instance, Joseph et al. [40], in their narrative review, concluded that “current evidence suggests that biases held by students remain consistent and may increase during healthcare education”. Similarly, Harris et al. [41] found that medical students in New Zealand showed a preference for New Zealand European patients over Māori patients in both vignette and IAT assessments. Haider et al. [42] reported that first year medical students in the United States demonstrated implicit preferences for white individuals and those from higher socio-economic backgrounds, although these biases did not always manifest in vignette-based decisions. One interpretation is that students may rely on deliberate, slow-thinking processes (System 2-thinking, in the terms of Kahneman [43]), allowing them to control or override unconscious biases in clinical decision-making scenarios. Interventions such as prejudice habit-breaking strategies may further mitigate bias in these contexts [44].

In contrast, studies involving more experienced healthcare professionals show a stronger association between IAT scores and clinical behaviour. Green et al. [21], for example, found that higher levels of racial bias was linked to a lower likelihood of prescribing thrombolysis to Black patients and a higher likelihood of treating White patients. Similarly, a longitudinal study by Van Ryn et al. [24] suggested that exposure to negative role-modelling by clinical educators was associated with increased bias in medical students over time.

One possible explanation for our findings may lie in the composition of our sample, which was predominantly female (91%) and different health professions yet no medical students. Prior research has shown that female healthcare professionals tend to exhibit lower levels of unconscious bias than their male counterparts [11, 45]. A more balanced gender distribution may have produced different results.

Another explanation could be generational change. Charlesworth and Banaji [46] reported declining levels of unconscious bias in the general population over recent years. This trend may also be reflected in health care students, most of whom in our sample were under the age of 23. Supporting this, Liang et al. [47] found that older health care professionals showed higher levels of implicit bias compared to younger ones.

A further possible explanation is, that there is not one traditional minority group in Switzerland with stereotypes regarding health or health behaviour. Rather, such stereotypes often only arise due to a lack of language skills. These in turn cannot be adequately conveyed in a written vignette.

Finally, institutional and curricular factors may have played a role. At our university, key elements of the curriculum, such as interprofessional training and value-based communication, are delivered across all degree programmes. These shared learning environments may contribute to a common understanding of professional values, thereby reducing differences across disciplines. In addition, the strong gender imbalance in our sample may have attenuated measurable effects. Future research including institutions with more gender-diverse student populations may yield more differentiated results, as other studies have suggested.

### Methodological considerations and limitations

To our best knowledge, this is the first study with students of health professions in Switzerland assessing unconscious bias towards patients using both vignettes and an IAT. It is also one of a few studies focussing on health professions beyond medicine and nursing and thereby addressing bias related to intersecting identities and contributing to the limited evidence base in the European context. In addition, the study adds to the understanding of whether such biases in health professionals may originate. Although our cross-section design does not allow causal conclusions, we found no indication that such biases emerge or become reinforced during professional training.

It is possible that the instruments used were not sensitive enough to detect subtle biases. While the vignette method is well established and considered valid, reliable, and practical [48, 49], it may not always elicit real-life behaviour. Although three-quarters of students rated the vignettes as realistic, it remains unclear whether the described situations led to authentic response patterns. Moreover, as the analyses were conducted at the group level (e.g., comparing semester or gender groups), potential individual-level variability in bias may not have been captured. The IAT is widely used but has also been criticised for limited predictive validity [50]. Nonetheless, other studies confirm its validity and reliability in measuring implicit bias [51]. In our study, the IAT showed no overall preference for heterosexuality or homosexuality, aligning with the explicit attitudes reported by students.

Our study has several limitations. First, data were collected at a single institution in the German-speaking part of Switzerland and is therefore not representative for whole of Switzerland. Second, the sample was predominantly female, which may have influenced results. Third, participation among students in later semesters was relatively low, possibly due to competing demands near graduation.

Importantly, our study used a cross-sectional design. While we compared early- and late-stage students, longitudinal data would offer more conclusive insights into how bias develops over time. We plan to follow up with the same cohort using personal codes to explore changes in implicit and explicit bias at the end of their programmes. Additionally, we have no control group from the general population. We therefore don’t know whether more altruistic students participated in the study or more altruistic young people choose a health care profession.

### Implications for education and practice

Our findings suggest that students in healthcare professions in this sample may begin their training with relatively egalitarian attitudes, and that professional education in the studied setting does not appear to foster unconscious bias. While this is encouraging, it should be interpreted with caution, as previous research has shown mixed results regarding the influence of professional education on bias formation [40]. Nevertheless, these findings may reflect the value-based curricula, interprofessional training, and increased societal emphasis on diversity and inclusion in the studied programs.

However, the findings raise new questions. Do students with more egalitarian values self-select into health professions? A comparison to the general population in Switzerland could help to explore this further. It is also possible that students’ attitudes change over time, with biases potentially becoming more pronounced as students transit into professional roles. To address these issues, a longitudinal study design following students throughout their education and into clinical practice would be valuable. Such an approach could shed light on the stability or development of both implicit and explicit biases over time and help to identify critical periods for educational intervention.

## Conclusions

This study found no evidence of unconscious bias in the helping intentions or IAT responses of students in health professions. Explicit and implicit attitudes were broadly aligned, and no bias was detected across key patient characteristics. While our cross-sectional design dowes not allow conclusion about how such biases develop, no indications emerged that they are acquired or reinforced during the course of study. These results offer a hopeful perspective on the next generation of health professionals, who appear to enter their careers with open-minded and inclusive attitudes. This gives hope despite the current backlash in numerous countries against including diversity, intersectionality, and inclusion, all of which are essential to reach a high standard of care for all patients, and thus counterbalances these ongoing challenges.

## Supplementary Information

Supplementary Material 1.

## Data Availability

The dataset generated from the current study is not publicly available due to data protection. However, on reasonable request and after a confirmation of the correct handling of data, especially in the context of the GDPR, an anonymized dataset can be made available by the corresponding author.
