# Supplementary material for: Unconscious bias in students of health professions – an experimental vignette study
Source: BMC Med Educ. 2025 Dec 10;26:65. doi: 10.1186/s12909-025-08317-x (PMC12801684; doi:10.1186/s12909-025-08317-x)
Supplement: Supplementary file 1 — Supplementary Material 1. [file 12909_2025_8317_MOESM1_ESM.docx]

Supplementary Material 1:
Figure 4: Pooled vignettes’ assessment interactions (intersectionality). Effect estimates with 95%-CI.

Supplementary Material 2:

Figure 5: Pooled vignettes’ assessment interactions (intersectionality). Margins (predicted means) of all combinations. Estimates with 95%-CI.
